# Environmental Enrichment and Successful Aging

**DOI:** 10.3389/fnbeh.2018.00155

**Published:** 2018-07-23

**Authors:** Michael Leon, Cynthia Woo

**Affiliations:** Department of Neurobiology and Behavior, University of California, Irvine, Irvine, CA, United States

**Keywords:** cognitive loss, cognitive impairment, cognitive enhancement, hearing impairment, mastication impairment, vision impairment, olfactory impairment, exercise

## Abstract

The human brain sustains a slow but progressive decline in function as it ages and these changes are particularly profound in cognitive processing. A potential contributor to this deterioration is the gradual decline in the functioning of multiple sensory systems and the effects they have on areas of the brain that mediate cognitive function. In older adults, diminished capacity is typically observed in the visual, auditory, masticatory, olfactory, and motor systems, and these age-related declines are associated with both a decline in cognitive proficiency, and a loss of neurons in regions of the brain. We will review how the loss of hearing, vision, mastication skills, olfactory impairment, and motoric decline accompany cognitive loss, and how improved functioning of these systems may aid in the restoration of the cognitive abilities in older adults. The human brain appears to require a great deal of stimulation to maintain its cognitive efficacy as people age and environmental enrichment may aid in its maintenance and recovery.

## Introduction

In more than a dozen animal models of human neurological pathology, environmental enrichment has been shown to ameliorate the human-like neurological symptoms present in these animals models, including the cognitive decline in aging (Segovia et al., 2006; Patel, 2012) and in Alzheimer’s disease (Arendash et al., 2004; Jankowsky et al., 2005; Lazarov et al., 2005; Berardi et al., 2007; Costa et al., 2007; Basak et al., 2008; Arranz et al., 2011; Polito et al., 2014).

Humes et al. (2013) suggested that the multi-sensory decline that accompanies normal aging in humans might influence the decline in cognition. They systematically examined changes in hearing, vision, touch, and cognitive function, in young, middle, and older adults. By varying the difficulty and sensitivity of the tasks, as well as making allowances for deteriorating sensory skills, they not only were able to show that age, global sensory, and global cognitive abilities were correlated, but also they further suggested that the decline in sensory processing and acuity may affect the cognitive decline. We present here a brief summary of the deterioration of the sensory and cognitive systems that occurs in older adults and we will present evidence that suggests that enriching the environment of the aging brain by reversing the decline in sensory systems may aid cognitive functioning.

## Hearing

### Neural Deterioration With Hearing Loss

Age-related hearing loss is common in older adults, with almost two-thirds of those over 70 years old having compromised hearing, and about a third of older adults experiencing debilitating hearing loss (Lin et al., 2011b; WHO, 2018). The odds of hearing loss are 5.5-fold higher in men than in women (Agrawal et al., 2008) and hearing sensitivity declines two times faster in men than in women, with diminished sensitivity observed in males as early as age 30 years (Syka, 2002).

Changes in brain anatomy accompany the decrements in auditory functioning. A reduction in the volume of gray matter is observed bilaterally in auditory cortex that correlates with the loss of high-frequency age-related hearing loss (Peelle et al., 2011; Eckert et al., 2012). There also are neural losses in areas that do not process auditory information directly. Husain et al. (2011) found that older adults with hearing loss experienced decreases in the volume of gray matter in the superior and medial frontal gyri, both of which are involved in cognitive processes, along with losses in the superior temporal cortex, which includes the auditory cortex and multisensory association cortex, and right anterior cingulate cortex, which includes both emotional and cognitive areas. Wong et al. (2010) found that older adults who had limitations in their ability to perceive speech in a noisy environment had smaller frontal cortices than younger adults with normal auditory ability.

In a longitudinal study over 6 years, Lin et al. (2014) compared whole-brain volumes of older adults with hearing impairment to those without hearing loss and found that those with hearing loss had an accelerated loss of brain tissue. There were also volume losses in the right temporal lobe, which included the superior, middle, and inferior temporal gyri, and the parahippocampus, areas that also mediate cognitive processes.

As for functional changes in the brain in response to auditory signals in hearing-impaired individuals, differences in evoked responses correlated with hearing impairment in noisy environments (Campbell and Sharma, 2013). In noisy environments, older adults with hearing loss also respond to speech with both a reduction in the activity of auditory brain areas and an increase in the activity of parietofrontal brain centers (Wong et al., 2010). In general, additional cognitive resources are recruited to achieve auditory perception under challenging auditory conditions for older adults (Eckert et al., 2008; Harris et al., 2009; Erb and Obleser, 2013; Erb et al., 2013; Vaden et al., 2013, 2016).

### Cognitive Deterioration With Hearing Loss

There is a consistent finding that has been revealed in five recent meta-analyses that hearing impairment in older adults increases the risk of cognitive deficiencies, including dementia (Thomson et al., 2017; Wei et al., 2017; Zheng et al., 2017; Loughrey et al., 2018; Yuan et al., 2018). Indeed, the risk of cognitive loss increased by 29% compared to normal-hearing older adults over the course of follow-ups of up to 6 years and that risk was increased by 57% when the patients were followed for a longer period, even when potential confounding factors were considered (Yuan et al., 2018). Moreover, increased peripheral damage of the auditory system predicted a greater risk of cognitive impairment in older adults (Yuan et al., 2018). Older adults with mild to severe hearing impairment have a two- to five-fold increase in their risk of developing dementia compared to older adults with normal hearing (Lin et al., 2011a). These cognitive impairments lead to a decreased ability to care for themselves, as confirmed by the impaired performance on the activities of daily living scales in older adults with hearing loss (Gopinath et al., 2013).

There are a number of ways that hearing impairment could affect cognition. The first is that there is a common mechanism that affects both neural systems independently, such as a gradual circulatory impairment, although the predictive value of hearing impairment appears to occur years before the cognitive decline (Albers et al., 2015). Another possibility is that the loss of hearing makes it difficult to have social interactions and increases the risk of depression, both of which could negatively impact cognitive ability (Mick et al., 2014; Dawes et al., 2015). Also possible is that as more effort is required for the listening process, fewer resources may be available for comprehension and memory, whereas the easing of the listening effort results in improvements in cognitive performance (Pichora-Fuller, 2006). Finally, it may be that the loss of auditory stimulation deprives the cognitive areas of their normal level of stimulation and thereby allows those areas to deteriorate.

### Enhanced Auditory Stimulation and Cognitive Function

If the loss of auditory stimulation allows the brain’s cognitive systems to deteriorate, would either the use of hearing aids or cochlear implants reverse the cognitive loss? Indeed, many studies have shown that the use of hearing aids is associated with improved cognitive abilities and decreased depression in older adults who have hearing loss (Mulrow et al., 1990; Tesch-Römer, 1997; Acar et al., 2011; Choi et al., 2011; Dawes et al., 2015; Castiglione et al., 2016; although see van Hooren et al., 2005).

Hearing aids, however, simply amplify and filter sounds and do not recreate the lost frequencies for their wearers. On the other hand, cochlear implants, even in older adults, restore a significant portion of the sound range that had been lost to the individual (Williamson et al., 2009; Carlson et al., 2010). Such implants restore not only auditory function, but also restore social interactions and quality of life for hearing impaired older adults (Olze et al., 2012). In addition, the cochlear implants induced improvements in cognitive function. Specifically, 12 months after cochlear implantation plus auditory training, 81% of the subjects with the lowest cognitive scores had improved their cognitive function (Mosnier et al., 2015). In another study of cochlear implantation in older adults with hearing impairment, the cochlear implants resulted in improvements in 70% of all cognitive tests, although the subjects did worse on 25% of the cognitive tests after surgery (Cosetti et al., 2016). Social isolation and depression also improved in those with auditory rehabilitation (Castiglione et al., 2016).

Interestingly, enhanced auditory enrichment started early in life also appears to impart benefits to cognition in later life. Lifelong music training appears to ameliorate the cognitive decline observed in older adults (Hanna-Pladdy and MacKay, 2011; Parbery-Clark et al., 2012; Zendel and Alain, 2012). Moreover, older adults with musical experience have enhanced ability to process speech (Bidelman and Alain, 2015). White-Schwoch et al. (2013) further showed that even moderate amounts of early music training had a persistent effect on maintaining the cognitive ability of older adults, even with a lapse of decades since they had played their instrument.

## Vision

### Visual and Cognitive Deterioration in Older Adults

Vision is compromised with age (Klaver et al., 1998; Muñoz et al., 2000; Salvi et al., 2006) and there is an increased risk of cognitive loss, Alzheimer’s disease and an increased clinical severity of Alzheimer’s disease among those with visual impairment (Uhlmann et al., 1991; Lin et al., 2004; Reyes-Ortiz et al., 2005; Whitson et al., 2010, 2012; Ong et al., 2012; Chen et al., 2013, 2017; Mine et al., 2016). Indeed, older adults with good visual acuity have a 63% decreased risk for developing dementia over an 8.5-year period and older adults with diminished visual acuity are 5 times more likely to experience cognitive loss than are older adults with good vision (Rogers and Langa, 2010). In addition, older adults with age-related cataracts have a 1.4 times increased likelihood of developing Alzheimer’s disease (Lai et al., 2014). It is also the case that older adults with poor vision engage in fewer activities that involve their cognitive ability than those with normal vision (Varin et al., 2017).

It is possible that there is a common mechanism that causes both vision and cognition to deteriorate. In fact, Drobny et al. (2005) found that older adults with poor vision did poorly on tasks that did not involve vision, suggesting that general cognition is deteriorating as vision deteriorates. When Dickinson and Rabbitt (1991) simulated visual impairment in normal subjects, the subjects were able to read the passage accurately, but their recall of material suffered, suggesting that the difficulty in storing the memory interfered with its retrieval. The cognitive loss that accompanies visual loss therefore may be due to such a situation.

### Neural Deterioration With Compromised Vision

Older adults with macular degeneration causing their visual impairment have a smaller visual cortex, along with a thinner cortex, smaller surface area, and lower gray matter volume than older adults with normal vision (Hernowo et al., 2014; Prins et al., 2016). Chen et al. (2013) found deterioration of gray matter in the visual cortical areas in those individuals with open-angle glaucoma, along with finding increased gray matter in some cognitive areas, including the medial area of the superior parietal cortex. The size of the frontal lobe and other areas involved in cognition decreases in older adults with macular degeneration, along with deterioration of the connections between visual cortex and frontal cortex, suggesting a cognitive link in this situation (Chen et al., 2013; Hernowo et al., 2014).

Blind individuals also have a significantly larger anterior right hippocampus compared to that of normal sighted individuals, and the posterior right hippocampus is significantly smaller in blind individuals (Leporé et al., 2009), suggesting modifications to regions of the brain involved with cognitive processing in response to diminished visual information.

### Compromised/Rectified Visual Stimulation, Cognitive Ability, and Visual Cortex

Wearing reading glasses is associated with improved cognitive outcomes for older adults (Spierer et al., 2016), although this condition could simply be the result of an increased ability to read. Indeed, the difference between groups was diminished when education level was considered. Unilateral cataract surgery in older adults can result in improved visual acuity, and that surgery improves cognitive functioning (Tamura et al., 2004; Ishii et al., 2008; Miyata et al., 2016), or maintains cognitive functioning (in comparison with other groups, which showed worsened cognitive functioning across the duration of the study; Elliott et al., 2009). It should be noted, however, that Anstey et al. (2006) did not find cognitive benefits after cataract surgery. Jefferis et al. (2015) found a small improvement in cognition after cataract surgery that was not correlated with improvement in visual acuity.

Older adults with visual impairments have a decrease in the volume of their visual cortex (Boucard et al., 2009) and unilateral cataract surgery can result in both an improvement in vision and an increase in visual cortex gray matter contralateral to the operated eye (Lou et al., 2013). Lin et al. (2018) found both improvements in visual acuity, cognitive functioning, and brain activity following bilateral cataract surgery, and these improvements were accompanied by an increase in gray matter volume in visual cortex, 6-months post-surgery.

### Combined Hearing and Visual Impairment

Diminished functionality in both vision and hearing senses, or dual sensory impairment (DSI), is a common problem in the older adult population, with greater than 70% of individuals with major vision impairments also exhibiting hearing loss (Heine and Browning, 2002). Individuals with both visual and hearing impairment have an even higher risk of cognitive loss, a diminished capacity on the daily living scales measures, an increased incidence of depression, a greater likelihood of social isolation, an increased risk of falls, and an increased risk of mortality (Keller et al., 1999; Saunders and Echt, 2007; Gopinath et al., 2013, 2016; Mitoku et al., 2016). Indeed, when combined, visual and auditory acuity account for 49.2% of the total variance and 93.1% of the age-related variance in intelligence in older adults (Lindenberger and Baltes, 1994). Both the high prevalence and the detrimental effects of DSI in the older adult population have resulted in recommendations for treatment that include mechanical aids to improve both hearing and vision deficits (Saunders and Echt, 2007; Zhou and Faure Walker, 2015; Gopinath et al., 2016).

## Olfaction

### Neural Deterioration With Olfactory Loss

Normal human aging is accompanied by a deterioration of olfactory abilities (Hoffman et al., 2016; Dong et al., 2017; Seubert et al., 2017), with 18% of older adults having a significant olfactory impairment and 46% of those over 80 years old having very limited olfactory ability (Hoffman et al., 1998; Murphy et al., 2002; Doty et al., 2015; Toussaint et al., 2015; Liu et al., 2016).

Differing from other sensory systems, which have cortical projections that are gated by the thalamus, the olfactory system has direct projections to brain regions involved with cognition and the loss or compromise of the olfaction system results in volume losses to many of these cortical brain areas in humans at any age (Bitter et al., 2010a,b, 2011; Peng et al., 2013; Shen et al., 2013; Yao et al., 2014). Olfactory projection sites, which include primary and secondary sensory cortical areas, as well as cortical regions involved with cognitive processing, also deteriorate with age (Segura et al., 2013; Kollndorfer et al., 2015).

### Olfactory System Deterioration and Cognitive Loss

Olfactory dysfunction also accompanies or precedes the early symptoms of cognitive disorders such as Alzheimer’s disease, Parkinson’s disease, Lewy body dementia, fronterotemporal dementia, Creutzfeldt-Jakob disease, mild cognitive impairment, and schizophrenia (Doty et al., 1988; Devanand et al., 2000; Ponsen et al., 2004; Tabaton et al., 2004; Ross et al., 2006; Luzzi et al., 2007; Wattendorf et al., 2009; Devanand et al., 2010; Li et al., 2010; Meusel et al., 2010; Nguyen et al., 2010; Parrao et al., 2012; Sohrabi et al., 2012; Conti et al., 2013). Given that these cognitive disorders have widely differing etiologies, it raises the possibility that the loss of olfactory stimulation contributes to the decline in cognitive ability in some of these disorders, particularly those in which olfactory loss precedes cognitive loss. Moreover, a degradation of olfactory ability predicts an elevated risk of mild cognitive impairment (MCI) and the degree of olfactory degradation may be used to predict which individuals with MCI will develop Alzheimer’s disease (Devanand et al., 2000; Peters et al., 2003; Schubert et al., 2008, 2017; Roberts et al., 2016; Lafaille-Magnan et al., 2017; Adams et al., 2018).

### Enhanced Olfactory Stimulation

Increasing olfactory stimulation in individuals who have experienced olfactory loss due to a variety of problems, such as post-infectious olfactory dysfunction, head trauma, Parkinson’s disease, and aging, has been shown to improve olfactory identification, olfactory discrimination, and to a lesser extent, olfactory thresholds (Hummel et al., 2009; Haehner et al., 2013; Konstantinidis et al., 2013; Damm et al., 2014; Geißler et al., 2014; Patel et al., 2017). These results were achieved using twice daily fragrance exposures to four odorants taken from each of four odor groups: resinous (eucalyptus), flowery (rose), fruity (lemon), and aromatic (clove) and the individuals continued this regimen for varying durations, (i.e., 12 weeks to 6 months). Further improvements in olfactory ability were observed with increased duration of exposure, increased concentration of the odorants, or an increased number of odorants (Damm et al., 2014; Altundag et al., 2015; Konstantinidis et al., 2016). A recent review and meta-analysis by Pekala et al. (2016) concluded that olfactory training was effective in improving olfactory system function (e.g., odor discrimination and identification), and positive results were observed following the loss of olfactory function with different etiologies. In addition to improvements in sensory ability, older adults exposed to increased olfactory stimulation have an improvement in their cognitive function, as evidenced by increased verbal fluency, an improvement in their depressive symptoms, and an improved sense of wellbeing (Birte-Antina et al., 2018).

## Mastication

### Cognitive Deterioration With Dental Problems

There are hundreds of reports that have examined the relationship between mastication (chewing) and cognition. Researchers have found a strong correlation between cognitive decline and poor oral health (for reviews, see Chen et al., 2015; Azuma et al., 2017). For example, there are a number of studies showing that the fewer teeth an individual has, the worse their cognitive ability is (Bergdahl et al., 2007; Stein et al., 2007; Syrjälä et al., 2007; Grabe et al., 2009; Okamoto et al., 2010; Lexomboon et al., 2012; Del Brutto et al., 2014; Mummolo et al., 2014; Peres et al., 2014; Elsig et al., 2015; Luo et al., 2015; Stewart et al., 2015). Contributing factors that can lead to tooth loss are periodontal disease (Noble et al., 2009; Kamer et al., 2012; Gil-Montoya et al., 2015; Welmer et al., 2017), untreated tooth caries (Tonetti et al., 2017), and low socioeconomic scale (Cabrera et al., 2005; Matsuyama et al., 2017). The subsequent consequences of tooth loss include increased stress levels (Budtz-Jørgensen, 1980), decreased social interactions, lower quality of life (Griffin et al., 2012), and a limited diet (Walls et al., 2000; Spaccavento et al., 2009; Kimura et al., 2013). All of these factors may be involved in the degradation of cognition, making it difficult to conclude that chewing supports cognition directly.

On the other hand, when rats and mice have teeth removed, or are given only powdered or liquid food to eat, their cognitive ability also declines (Kato et al., 1997; Yamamoto and Hirayama, 2001; Fukushima-Nakayama et al., 2017), along with a decrease in brain-derived neurotrophic factor (BDNF), synaptic density, and neuronal number in the hippocampus (Onozuka et al., 1999; Yamamoto and Hirayama, 2001; Okihara et al., 2014; Takeda et al., 2016). In addition, there is widespread volumetric loss of gray matter in the brain following tooth loss in mice (Avivi-Arber et al., 2016). At the same time, disruption of normal chewing leads to elevated levels of corticosterone (Kubo et al., 2007) and lower levels of hippocampal glucocorticoid receptors (Ichihashi et al., 2008) that are associated with chronic stress (Sapolsky et al., 1984). The advantage of examining chewing activity in non-human species is that the caloric intake and amount of food can be equalized across the hard and soft food eating conditions, thus maintaining similar nutritional values. The results of these animal studies support the idea that the active process of chewing plays a role in cognitive functioning and stress reduction. Indeed, in humans, a stronger correlation was observed between chewing ability (with or without the use of dental prostheses) and cognitive function than that observed between tooth loss and cognition (Lexomboon et al., 2012).

### Enhanced Mastication/Corrected Dentition and Cognitive Restoration

While the loss of normal chewing ability is associated with cognitive decline, increased chewing activity has been shown to decrease stress and improve cognition in humans (Baker et al., 2004; Stephens and Tunney, 2004; Scholey et al., 2009; Ono et al., 2010; Yu et al., 2013; Azuma et al., 2017; for a review, see Weijenberg et al., 2011). Chewing gum alone can increase attention, decrease reaction times, and improve mood, even under stressful conditions (Smith, 2010; Kubo et al., 2015), as well as increasing cognitive processing speed (Hirano et al., 2013). A meta-analysis found positive effects of chewing on alertness or attention in 64% of the studies they examined (Hirano and Onozuka, 2015). In contrast, Tucha et al. (2004) observed chewing gum only changed aspects of attention without cognitive improvement. Consistent with the notion that chewing may aid cognition, the brain areas that are activated by chewing include cognitive centers such as the frontal cortex and the medial temporal lobe (Onozuka et al., 2002; Choi et al., 2017).

Rats and mice given wood dowels to chew have a reduced stress response and are able to maintain their hippocampal-dependent cognitive function (Ono et al., 2010; Miyake et al., 2012; Chen et al., 2015). In addition, the chewing action relieves the stress-induced suppression of cell proliferation in the hippocampal dentate gyrus that may underlie the impaired hippocampal functioning with tooth loss (Kubo et al., 2007). Also, switching mice from powdered food to hard pellet food reverses the suppression of neurogenesis in the forebrain subventricular zone that occurred while on the soft food diet (Utsugi et al., 2014).

Humans who have lost teeth and were subsequently given dentures or dental implants that restored dental function also had improved prefrontal cortex activity (Narita et al., 2009; Kimoto et al., 2011; Kamiya et al., 2016), along with improved cognitive performance (Cerutti-Kopplin et al., 2015; Banu et al., 2016; De Cicco et al., 2016).

## Sensorimotor/Somatosensory Stimulation

### Exercise Increases/Restores the Size of Cortical Structures

Decreases in the size of the brain, including the hippocampus, are observed in normally aging older adults, and this decrease accompanies the declines in cognitive functioning (Raz et al., 2005). Aerobic exercise can restore some of these decreases in brain volume. About 12 months of aerobic exercise resulted in an increase in the volume of the hippocampus (Erickson et al., 2011, 2014; Niemann et al., 2014) and 6 months of aerobic training increased the volume of gray and white matter, while stretching and toning for the same amount of time did not result in brain volume changes (Colcombe et al., 2006). In addition, both hippocampal volume and dorsolateral prefrontal cortex thickness were positively correlated with aerobic fitness (Jonasson et al., 2017).

### Cognitive Benefits of Exercise

Multiple studies, reviews, and meta-analyses have concluded that in older adults, exercise training results in improvements in cognitive skills, along with improved health and mobility (Colcombe and Kramer, 2003; Hertzog et al., 2008; Kemoun et al., 2010; Bherer et al., 2013; Kirk-Sanchez and McGough, 2014; Barha et al., 2017; Gregory et al., 2017; Kennedy et al., 2017; Mavros et al., 2017; Saez de Asteasu et al., 2017; Northey et al., 2018; for review see Erickson et al., 2013). In addition, following exercise training, improvements in cognitive functioning are correlated with measurable increases in brain volume (Erickson et al., 2011; Mortimer et al., 2012) and increased BDNF levels (Erickson et al., 2011; Sungkarat et al., 2018), both of which may underlie the observed cognitive gains (Erickson et al., 2013). Exercise training also restores the decrease in interhemispheric inhibition typically observed in older adults, which could lead to improved motor control (McGregor et al., 2018). In addition, using fMRI to measure brain activity, exercise for 12 months improved functional connectivity among the cortices and the improved connectivity was associated with improved executive function (Voss et al., 2010). Moreover, adults who exercised showed altered activity in the right inferior frontal gyrus during a semantic verbal fluency task that was correlated with improvements in the task (Nocera et al., 2017). A recent meta-analysis that examined the influence of exercise, cognitive training, or the two combined on falls and cognition in older adults with MCI found that exercise or the combination of exercise and cognitive training resulted in gait speed and balance improvements, as well as cognitive function gains, all of which can contribute to decreased incidence of falls (Lipardo et al., 2017).

### Mechanisms by Which Exercise Can Improve Cognition

Stimpson et al. (2018) have proposed a model for the mechanism underlying cognitive improvement following exercise. Specifically, they first note that exercise increases cerebral angiogenesis and circulation in the brain. Pereira et al. (2007) reported that after 3 months of aerobic exercise, there was increased neurogenesis and increased cerebral blood volume in the human hippocampus, raising the possibility that increased angiogenesis mediated these improvements. There is also a transient elevation of serum BDNF after humans exercise (Ferris et al., 2007; Håkansson et al., 2017), and when the action of BDNF is blocked in rats after exercise, the cognitive benefits are also blocked (Vaynman et al., 2004). They next noted that exercise also decreases chronic inflammation in older adults (Cotman et al., 2007) and that restores elevated levels of insulin-like growth factor (IGF-1), which elevates BDNF levels and increases neurogenesis (Carro et al., 2000). The increased neurogenesis then allows the improved cognitive outcomes following exercise. It should be noted, however, that the presence of adult human neurogenesis has been recently questioned (Sorrells et al., 2018).

It is also the case that a relatively recent meta-analysis on studies showing aerobic gains with exercise came to the conclusion that there was no compelling evidence indicating that exercise improves cognitive abilities in older adults (Young et al., 2015). If increased aerobic capacity does not drive the effects of exercise, then it seems possible that the increase in somatosensory stimulation that is experienced during exercise may drive any improvements that are seen following exercise. This perspective would then bring any cognitive improvements with exercise in line with the effects of other types of sensory stimulation that we have discussed.

## Conclusion

### Deterioration of Sensory Systems Can Be Mitigated

It seems clear that the deterioration of sensorimotor systems contributes to the decline in cognition seen in older adults, either directly, by providing less stimulation to the cognitive areas of the brain, or indirectly, by depriving older adults of the nutrition, intellectual engagement, or social engagement that they need to thrive. Currently, there are methods that attempt to restore the declining systems, and although they are not always able to completely restore sensory system function, they do appear to aid individuals, with improvements to sensory acuity, cognition, and quality of life, thus improving chances of successful aging. Importantly, it is apparent that early detection of deficits to any of these systems is critical, and restoration or repair of any deficits should be prioritized to increase the likelihood of maintaining cognitive and full body health. Most certainly, new technologies are needed to continue to refine and improve the restoration of individual sensorimotor systems. Additional environmental enrichment using improvements to mastication or exercise training results in cognitive and health gains, which may potentially further aid in the successful aging of older adults.

### Barriers to Maintenance or Restoration of Cognition Function by Restoring Deteriorating Sensory Systems in Older Adults

Although the aids described previously for use in the restoration of sensory and motor systems are promising, the benefits are not without a cost and may not be experienced by everyone. While it makes sense to do what one can to sustain or repair these systems, the cost of such repairs is not affordable by most people. Perhaps the greatest need is in hearing aids because a set can cost thousands of dollars and these devices are typically not covered by medical insurance in the United States. Consequently, only 3–4% of those with mild hearing loss who need hearing aids are wearing them (Chien and Lin, 2012). Cochlear implants, while even more expensive, can be covered by medical insurance, but are called for only in those with severe hearing impairments and in deaf individuals (Lin et al., 2012). The use of reading glasses seems to be a useful tool to allow older adults to engage with their environment effectively. While some investigators find a cognitive benefit for cataract surgery, others do not observe such an improvement, suggesting that the cognitive benefits may not be reliable. Dentures and dental implants are effective for maintaining or restoring cognitive ability for older adults with poor dentition, but again it comes at great expense, and it is something that Medicare does not cover. Enhanced olfactory stimulation has had limited tests for cognitive improvement and there are no studies showing improved neural responses after enhanced olfactory stimulation in older adults.

It is difficult to induce older adults to exercise regularly. Only about 16% of older adults engage in the recommended amount of physical activity on a daily basis (Centers for Disease Control and Prevention [CDC], 2018), which are at least 150 min of moderate-intensity aerobic activity or 75 min of vigorous-intensity aerobic activity and 2 or more days of muscle-strengthening activities per week (Centers for Disease Control and Prevention [CDC], 2018). Indeed, 33% of older adults reported no physical activity at all (Centers for Disease Control and Prevention [CDC], 2018). Barriers to exercise include lack of motivation, lack of knowledge, pain, poor health, physical limitations, peer pressure, and bad weather (Costello et al., 2011; Gothe and Kendall, 2016). Surprisingly, older adults do not have an increased risk of injury from exercise (Stathokostas et al., 2013). Although there is a concern that the oxidative stress associated with intense exercise would hasten aging in older adults, physically active older adults actually have reduced exercise-induced oxidative stress than older adults with a lower level of physical activity. Additionally, regular physical activity apparently improves the antioxidant defenses of older adults (Meijer et al., 2002).

### The Possibility of a Common Factor

While each sensory system may have its impact on cognition in its own way, there is also the possibility that there is a common factor that underlies their role in the maintenance of cognitive processes. For example, the loss or degradation of each sensory system may result in an emotional change that could impact cognitive performance. As discussed above, depression is associated with the loss of teeth (Shah et al., 2015), is associated with the loss of olfactory stimulation (Kohli et al., 2016), and often follows the degradation of the auditory and visual systems. Depression also often follows cognitive dysfunction in older adults (Yin et al., 2015). Alternatively, the common factor could be a physiological element that deteriorates both sensory and cognitive brain systems concurrently, but deficits are more easily detectable in the sensory systems. It also may be that continual exposure to environmental elements may wear on the sensory systems before they impact the cognitive brain areas.

### Future Directions

One future direction may be to enhance stimulation of olfactory, visual, auditory, tactile, masticatory, and motor systems simultaneously or in conjunction. This type of sensorimotor stimulation had benefits for individuals with developmental neurological disorders (Woo and Leon, 2013; Woo et al., 2015; Aronoff et al., 2016). Environmental enrichment has also been shown to improve the symptoms of children with Rett syndrome (Downs et al., 2018). Similar forms of sensorimotor combination therapy may have health benefits for aging adults.

## Author Contributions

ML and CW conceived and wrote this review.

## Conflict of Interest Statement

The authors declare that the research was conducted in the absence of any commercial or financial relationships that could be construed as a potential conflict of interest.
